# Characteristics of Hospitalized Pediatric Patients in the First Five Waves of the COVID-19 Pandemic in a Single Center in Poland—1407 Cases

**DOI:** 10.3390/jcm11226806

**Published:** 2022-11-17

**Authors:** Lidia Stopyra, Aleksandra Kowalik, Justyna Stala, Ida Majchrzak, Justyna Szebla, Mateusz Jakosz, Karolina Grzywaczewska, Przemko Kwinta

**Affiliations:** 1 Department of Infectious Diseases and Pediatrics, Zeromski Specialist Hospital in Krakow, 30-931 Kraków, Poland; 2 Department of Pediatrics, Jagiellonian University Medical College, 30-663 Kraków, Poland

**Keywords:** COVID-19, SARS-CoV-2, children, pandemic, waves, hospitalization, clinical presentation

## Abstract

This is a single-center, prospective study that compared the clinical presentation and laboratory findings of hospitalized children during the first five waves of the COVID-19 pandemic. Data were collected, according to a standardized questionnaire, from 1407 children from 23 March 2020 to 30 April 2022. Significant differences in clinical courses were found among the five waves probably due to different SARS-CoV-2 variants. The median age was 95.8 months in the first wave versus 14.6–23 months in the others. The number of patients with upper respiratory infection was the highest in the fifth wave (74.4% versus 43.8–56.9% in the others) and for lower respiratory infection in the first wave (50.0% versus 16.4–32.5%). Gastroenterocolitis was more common in the fifth wave (24.4% versus 8.9–16.5%); neurological diagnoses appeared more frequently in the fourth wave (16.6% versus 0.6–9.9%), while anosmia and ageusia were higher in the fifth wave (13% versus 1.5–4%). Life-threatening courses were relatively rare. However, children with pneumonia, dehydration from high fever, gastrointestinal symptoms, loss of smell and taste, and neurological symptoms required hospitalization.

## 1. Introduction

In February 2020, the World Health Organization (WHO) designated a new strain of betacoronavirus as severe acute respiratory syndrome coronavirus 2 (SARS-CoV-2), the causative agent of coronavirus disease 2019 (COVID-19) [1]. Fever, cough, and dyspnea were initially indicated as the presenting symptoms of SARS-CoV-2 infection. Severe pneumonia with respiratory failure reported on 31 December 2019 in the region of Wuhan, China, was the reason for hospitalization and life-threatening situations [2].

In the first reports of COVID-19, the frequency of disease in children appeared much lower than in adults. Both in China and in Italy, only 1% of cases were pediatric [2,3]. In the following months, the number of pediatric patients gradually increased. For instance, in the U.S., at the beginning of the pandemic, 2.2–4.2% of the reported cases were pediatric; then, according to reports from the American Academy of Pediatrics (AAP), the rate increased to 14.3% and in 10 states, children accounted for over 18% of cases [4,5]. Data from the European Centre for Disease Prevention and Control (ECDC) showed that up to 17.6% of cases were pediatric [6]. A lot of studies published have confirmed the clinical impression that COVID-19 in children typically presents as a mild (37%) or moderate (45%) upper respiratory tract infection and is rarely severe or critical [7]. Other signs and symptoms described in children include gastrointestinal, anosmia, ageusia, neurological, and dermatologic manifestations [2,3,7,8,9,10,11,12,13,14,15,16,17].

Numerous SARS-CoV-2 variants have circulated globally since the beginning of the pandemic, and differences in their courses have been reported [1,4,10,11,12,16,17,18,19,20,21,22,23,24,25,26,27,28,29]. The increasing number of pediatric cases and changing clinical disease presentations might require changes in COVID-19 management for children, risk group identification, testing criteria, and indications for hospitalization.

The aim of this paper was to describe the COVID-19 characteristics in hospitalized Polish children during the first five waves and to assess whether there were any differences among the different waves. In particular, the trends in the demographic data, clinical presentation, laboratory findings, and COVID-19 outcomes over two years of the pandemic were analyzed. Our observations may be useful for ongoing guidance for the evaluation, management, and prevention of COVID-19 in children.

## 2. Materials and Methods

Because of the WHO’s announcement of a pandemic and the increasing cases of COVID-19 in Poland, the Department of Infectious Diseases and Pediatrics was instituted to be the central unit for treating pediatric COVID-19 cases in the southern region. The first two children were admitted to hospital on 23 March 2020, which is when this study commenced. Every patient from 0 to 18 years of age with confirmed COVID-19 hospitalized between March 2020 and April 2022 was included.

Following the recommendations from the WHO and the National Institute of Public Health [30,31], COVID-19 was diagnosed using a positive reverse transcription and real-time polymerase chain reaction (RT-PCR) test. Since 30 October 2020, second-generation antigen tests from a nasopharyngeal swab were performed in certified laboratories. Several kits were used: (1) GeneFinder™ COVID-19 Plus RealAmp, Elitech, Biomedica (Oxford, UK); (2) Liferiver, Novel Coronavirus (2019-nCoV) Real Time Multiplex; (3) VIASURE CerTest, Biotec (Zaragoza, Spain); (4) Maccura SARS-CoV-2 Fluorescent PCR, Maccura Biotechnology (Sichuan, China); (5) Homemade DIAGtest SARS-CoV-2 real time RT-PCR; (6) Labsystems Diagnostics (Vantaa, Finland). COVID-19 Real Time Multiplex RT-PCR and the second-generation Abbott Panbio-COVID-19 Ag Rapid Test Device (WHO laboratory 2020, AOTM).

Criteria for hospital admission were similar to other pediatric infection diseases, such as dehydration from fever, vomiting, and diarrhea. According to the Polish Ministry of Health [32], hospitalization was compulsory for every patient with diagnosed SARS-CoV-2 infection up to September 2020. According to Polish expert group recommendations, hospital referrals were also required for children with congenital heart defects, neurologic diseases, genetic disorders, chronic renal diseases, mucoviscidosis, broncho-pulmonary dysplasia, immunodeficiency after organ transplantation, and diabetes mellitus. Included also were newborns, infants, and children with obesity, especially with a body mass index (BMI) >30 kg/m^2^ [33].

Discharge criteria were two negative PCR tests taken within 24 h. After 2 September 2020, the only criterion was the condition of the patient.

The disease severity assessment in this analysis was based on the need for oxygen, intravenous rehydration or steroids, and the length of stay. Antiviral therapy was also assessed. Systemic steroid and antiviral therapy were used according to the recommendations from the beginning of the pandemic [33,34,35,36,37,38,39] with the following changes: Dexamethasone was used according to the European Medicines Agency’s (EMA) recommendations in hospitalized patients, especially in those treated with remdesivir at a dose of 0.1 mg/kg for a maximum of 4 mg/24 h [34]. Dexamethasone was also used in some patients with laryngitis according to references from previous studies [40,41].

Remdesivir was used in our department according to the Food and Drug Administration’s (FDA) and EMA’s recommendations [35,42]. According to the product characteristics, remdesivir was used in patients 12 years of age and older weighing at least 40 kg. It was also used in pediatric patients weighing at least 3.5 kg with positive results for direct SARS-CoV-2 testing with pneumonia and requiring oxygen supplementation. Baricitinib was used in patients aged 2–18 years who required non-invasive or invasive mechanical ventilation with recommended dosages under the Emergency Use Authorization (EUA): for patients aged nine years or older, 4 mg once daily, and for those aged two to less than nine years, 2 mg once daily [43]. Data were collected and reported by the physicians working in the department according to a standardized case history questionnaire and a physical examination for every patient. Symptoms were recorded at the time of hospitalization. Standard laboratory tests were conducted for every child diagnosed with COVID-19.

All patients included in the study were symptomatic. The questionnaire included:

1. Demographic data: age, sex, ethnicity, recent contact with patients with COVID-19, and comorbidities (e.g., heart, chronic lung, neurological, or genetic diseases; asthma; developmental delay; diabetes; immunodeficiency, or malignancy).
2. Signs and symptoms: fever, cough, rhinitis, dyspnea, sore throat, weakness, diarrhea, abdominal pain, vomiting, headache, conjunctivitis, nausea, myalgia, rash, ageusia, anosmia, chest pain, or irritability.
3. Disease outcome data: length of hospitalization, complications, oxygen treatment, casual treatment, pediatric intensive care unit (PICU) admission, or death.
4. Laboratory data: complete blood count (CBC) parameters, C-reactive protein (CRP), alanine transaminase (ALT), lactate dehydrogenase (LDH), creatinine kinase (CK), ferritin, vitamin D3 level, prothrombin time, D-dimers, nasal swabs for other viral pathogens (co-infection), and imaging (i.e., lung ultrasound (LU), chest X-ray, and high-resolution computed tomography (HRCT)).
5. Final diagnoses: Upper or lower respiratory tract infection, gastroenterocolitis, or neurological diagnoses.

Lower respiratory infections were diagnosed based on clinical presentation and LU, chest X-ray, and HRCT. The examination taken most often, especially in the youngest children, was LU. The presence of focal, multifocal, and confluent B lines and pleural irregularities were the most common LU findings for diagnosing pneumonia from COVID-19. In chest X-ray examinations, bilateral and multifocal lesions were found most frequently, especially in the lower lobes. The pure ground-glass appearance was also typical for COVID-19 lower respiratory-related findings [44,45,46]. Regarding gastrointestinal infection, diagnosis was based on clinical presentation (i.e., vomiting or diarrhea) and the exclusion of any other etiology such as rotavirus, adenovirus, and norovirus.

Statistical analysis was performed using SPSS ver. 27 software (Armonk, NY, USA). The results are presented based on the parameters of descriptive statistics, including either the mean value and standard deviation (SD) for the quantitative variables with normal distribution or the median value with the interquartile range in the opposite case. Categorical variables are presented as numbers with percentages. Qualitative values were compared using the chi-squared test. For the analysis of continuous variables, a Kruskal–Wallis test was used. In all cases of statistical significance, a pairwise comparison between groups was performed using a post hoc test. In all of the analyses, *p* < 0.05 was considered statistically significant.

This study was performed in accordance with the ethical standards of the Declaration of Helsinki and its later amendments. It was approved by the Ethics Committee of the Regional Medical Chamber in Krakow, No. OIL/KBL/18/2020, on 10 March 2020.

## 3. Results

We compared the data characteristics of those children and adolescents admitted with acute COVID-19 during the first five waves of the pandemic.

### 3.1. Study Groups

**Figure 1:**
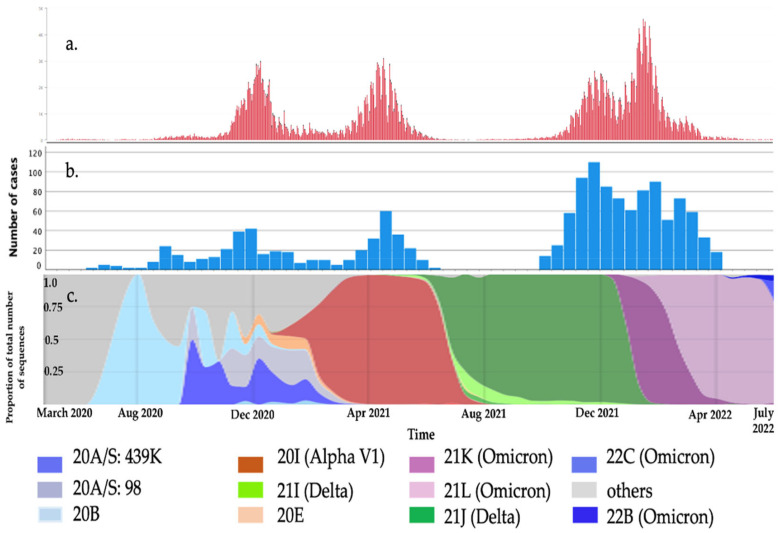
COVID-19 cases in our region: (**a**). Daily COVID-19 cases in the Malopolska region based on [47]. (**b**). Number of hospitalized children with COVID-19. (**c**). The proportion of the total number of SARS-CoV-2 variants over time in Poland based on [48].

This study comprised 1407 patients: 112 (8%) from the first wave (1 March to 30 September 2020); 175 (12.4%) from the second (1 October 2020 to 31 January 2021); 195 (13.8%) from the third (1 February to 31 May 2021); 511 (36.3%) from the fourth (1 October 2021 to 15 January 2022); 414 (29.5%) from the fifth (16 January to 30 April 2022) (Figure 1). All but one of the children were white European; the other was of Asian background.

Table 1 shows the demographic characteristics of the hospitalized patients.

In all waves, more boys than girls were hospitalized (from a low of 50.5% in the fourth wave to a high of 56.6% in the second), with no statistical significance between waves. The median age was the highest in the first wave (95.8 months) and significantly lower in others, decreasing in the following waves: 23 months in the second, 20.1 months in the third, 17.6 months in the fourth, and 14.6 months in the fifth (*p* < 0.001). Chronic comorbidities, which related to high risk of severe COVID-19 were present in 21.1% of patients in the second wave to 34.8% in the first, and there were no statistically significant differences in the comorbidity frequency between waves (*p =* 0.06).

### 3.2. Clinical Presentation

The clinical presentation of pediatric COVID-19 during the first five waves of the pandemic is shown in Table 2.

The most frequent symptom in all waves was fever. (68% in the fourth wave to 75% in the third), with no statistically significant differences between waves. The fever was defined as a temperature above 37.5 °C (99.5 °F) in axillary, ear, and forehead temperature measurements. In the case of respiratory symptoms, rhinitis was most frequently reported in the fourth wave (52% of patients), and significantly the least in the first (21%), whereas cough was most common in the third (61%) and fourth waves (64%) (*p* < 0.001). Dyspnea was a relatively rare symptom, although the study included only hospitalized patients—7.4% in the second wave to 14% in the fourth, with no statistically significant differences between waves.

In the case of gastrointestinal symptoms, vomiting was the rarest in the first wave (6.3%) and the most common in the fifth (30%) (*p* < 0.001), and diarrhea was the most common in the second wave (31% versus 18–25% in the others) (*p* = 0.002).

Anosmia and ageusia, the most specific COVID-19 symptoms, were rare in the children in the first four waves (1.5–4% of children), but the frequency of these symptoms was much higher in the fifth wave (13%) (*p* < 0.001).

Neurological manifestations (seizures and impaired coordination and balance) appeared in 4.6% of the patients in the third wave to 14% of the patients in the first wave, and the differences between waves were statistically insignificant. Table 2 shows the symptoms by wave.

### 3.3. Laboratory Findings

The laboratory findings from the children during the first five waves of the pandemic are shown in Table 3. There were no statistical differences between the groups at the CRP level or in the number of neutrophils, but there were differences between the waves in seven parameters. The number of leukocytes was the lowest in the first wave (median of 6.4 × 10^3^/μL) (*p* < 0.001), similar in the others (7.75–8.9 × 10^3^/μL). The number of lymphocytes was also the lowest in the first (median of 2.3 × 10^3^/μL vs. 3.49, 3.96, 3.61, and 3.39 × 10^3^/μL (*p* < 0.001) in waves 2–5, respectively). The first-wave patients also had the lowest platelet count (median of 247 vs. 309, 303, 279, and 281 × 10^3^/μL in waves 2–5, respectively (*p* < 0.001). There were also differences in alanine transaminase and creatinine kinase (*p* < 0.001), but the post hoc analysis revealed them to be significantly higher in the fifth wave.

The Kruskal–Wallis test showed significant differences between the groups in LDH level (*p* < 0.001); however, in the post hoc analysis, the first and fifth wave groups differed from the others. D-dimers were significantly lower in the first wave versus the second, fourth, and fifth waves (*p* < 0.001). In the chi-squared test, significant differences were found in the leukocyte (*p* = 0.003) and lymphocyte levels (*p* < 0.001), creatinine kinase (*p* = 0.005), and lactate dehydrogenase and D-dimers (*p* < 0.001).

### 3.4. COVID-19 Severity

The COVID-19 severity data are included in Table 4. Oxygen therapy was required in 0% (first wave) to 4% (fourth wave) of the patients and there were no statistically significant differences between the five waves (*p* = 0.071). Differences were found in the need for intravenous rehydration—most common in the fifth wave (59%) and least in the third wave (9%) (*p* < 0.001). Systemic steroid therapy was used the least in the second wave (1.1%) and the most in the fourth wave (11.2%) (*p* < 0.001). The length of stay was significantly shorter in the fifth wave (median of three days). The post hoc analysis revealed differences between the fifth and all other waves (*p* < 0.001). Only one (0.5%) patient in the third wave and two (0.3%) in the fourth were referred to a PICU, but no one died. One patient in our department needed high-flow nasal oxygen therapy (HFNOT). Nine (1.7%) patients in the fourth wave were treated with remdesivir (0.64% during the whole pandemic) and 1 (0.19%) with baricitinib according to FDA and EMA recommendations [25,30,31]. Two (0.4%) patients in the fifth wave were treated with baricitinib (0.21% during the whole pandemic).

### 3.5. Final Diagnoses

Because of the overlap, there were 1862 diagnoses in the 1407 patients, of whom 235 (16.7%) had more than one final diagnosis: urinary tract infection (UTI) combined with gastroenterocolitis, pneumonia and gastroenterocolitis, and upper respiratory tract infection and seizures or suicide attempts. In the first and second waves, there were 1.2 diagnoses per patient, but that increased in the following waves to 1.26 in the third, 1.35 in the fourth, and 1.39 in the fifth. The average for the whole period was 1.32. This means that through subsequent waves the symptomatology of COVID-19 in children was becoming richer.

The most common final diagnoses were upper respiratory, lower respiratory, and gastrointestinal infections (Table 5). Upper respiratory infections were the most common in the fifth wave (74.3%) and the least in the first wave (43.8%) (*p* < 0.001). Rhinitis and laryngitis were reported the most frequently. Lower respiratory infections were diagnosed based on clinical presentation and LU, chest X-ray, and HRCT. Lung imaging data from the children during the first five waves of the pandemic is shown in Table 6. It was the most common in the first wave (50%) and least common in the fifth wave (16.4%) (*p* < 0.001), whereas gastroenterocolitis was the most frequent in the fifth wave (24.4%) and the least in the first wave (8.9%). Significant differences were observed between the five waves in the frequency of neurological diagnoses, especially between the second (0.6%) and fourth (16.6%) waves (*p* < 0.001).

## 4. Discussion

To the best of our knowledge, this is the largest single-center study of children hospitalized due to COVID-19 and the first one comparing clinical presentations in children during the first five waves of the pandemic. Although children are considered to be less affected [12,18,19,49,50,51], 1407 were hospitalized between 23 March 2020 and 30 April 2022. This might have been due to the higher prevalence of SARS-CoV-2 in our local community and the central organization of hospital care in our region. The first wave of the pandemic was very mild in Poland because of the strict lockdown in the spring of 2020, which means that the relatively high number of hospitalized children in the first wave was the result of mandatory hospitalization for every infected SARS-CoV-2 patient [32] (Figure 1).

### 4.1. Demographic Characteristics

The demographic characteristics of the patients were similar in all five waves. There were no significant differences in sex, but there was a slight male predominance, as in other studies [15,16,20,50,52,53,54].

The ages of our patients were of particular interest. The median age in the first wave (95.8 months) was higher compared to the others (14.6–23 months). Similarly, infants aged zero to six months represented 26–29% of patients from the second to fifth waves. Other authors have reported the prevalence of both younger [2,15,16,17,54] and older children [8]. For example, Turan et al. [16] revealed the prevalence of younger children in the second wave compared to the first. It should be noted that, to the best of our knowledge, there has not been such a large study of the prevalence of children with COVID-19 at such a young age. This can be explained by outbreaks of COVID-19 in large neonatal departments and the referral to our department of children at risk of a severe course of COVID-19. The Polish expert group recommendations also indicate the necessity of hospitalizing the youngest children [33]. It is noteworthy that our study included only hospitalized children.

### 4.2. Clinical Presentation

Though SARS-CoV-2 infection was common in children, the course of the disease was usually milder than for adults [12,18,19,49,50,51]. In our department, severe courses of the disease were rare, and there were no significant differences in severity over the five waves, although we did observe increased hospitalizations in the fourth and fifth waves. Similar observations regarding increasing numbers of hospitalization for the delta and omicron variants were reported by Marks et al. and Shi et al. [21,22,23]. However, we found significant differences in their clinical presentations. Similar observations have been reported by other authors [9,13,14,24,25,26,27,28,33,50].

The basic differences in the clinical presentation were the frequency of respiratory symptoms (rhinitis, cough, dyspnea, auscultatory changes, and lower respiratory infection), which increased from the second to the fourth waves. In contrast, gastrointestinal symptoms (vomiting and diarrhea) were the most common in the second wave. Other authors have reported fever and cough as the most frequent early symptoms [9,10,11,12]. During the predominance of the delta and omicron variants, upper respiratory tract symptoms (rhinitis and sore throat) were more common [29].

Anosmia and ageusia, the most significant symptoms of COVID-19, were very rare in the children: Fewer than 4% of the patients in our study, which differed significantly from previous reports. Most authors have emphasized that anosmia and ageusia caused by the omicron variant appeared much less often in the fifth wave [29,55,56,57]. This might have been caused by the specific nature of our cohort—only hospitalized children, who showed a significant decrease in age from wave to wave (Table 1). In the fifth wave, the median age was 14.6 months. This was a special group of patients who might require hospitalization for dehydration resulting from the refusal to take fluids due to smell and taste disorders. In such cases, medical help was sought, as feeding the youngest children proved difficult. In older children and adults, smell and taste disorders did not usually require hospitalization. It is noteworthy that the results were also affected by the team’s increasing experience in COVID-19 diagnosis in the youngest group of patients, who were unable to verbalize their ailments. It is also worth emphasizing that some authors have reported the frequency of smell disorders in the fifth wave of the pandemic as 12% and taste as 23%, which was more frequent than in our cohort (13% in both cases) [58].

Regarding the final diagnoses of the hospitalized COVID-19 pediatric patients, the number of children with upper respiratory or gastroenterological symptoms was the highest in the fifth wave, while that of lower respiratory infection was most common in the first wave. Interestingly, Pokorska-Śpiewak et al. [12] reported in their study that pneumonia was more common in the second than in the first wave, but this can be explained by lower testing for SARS-CoV-2 infection of asymptomatic or mildly symptomatic children in our region during the first wave. We observed more upper than lower respiratory infections and shorter lengths of stay in hospital in the fifth wave. A lot of publications support our study’s finding of a milder course for the omicron-dominated fifth wave in both adults and children [59,60,61,62]. Marks and Shi reported that the proportions of hospitalized children requiring PICU or intensive mechanical ventilation were similar in the first four waves but lower in the fifth [21,22,23]. Nevertheless, although most of the patients who contracted the SARS-CoV-2 omicron variant exhibited milder clinical features, severe clinical features, including mortality, were encountered among individuals who were not vaccinated [63].

In our cohort, more neurological symptoms occurred in the fourth wave. Similarly, in London, Molteni compared the disease course during the alpha and delta variant predominance and found more neurological symptoms (headaches, dizziness, chills, anosmia, and ageusia) during the delta variant period [19].

Antoon et al., who analyzed only serious neurological complications and those of clear significance (seizures, strokes, and encephalopathy), also reported that the most common neurological diagnoses occurred in the delta variant period (37.8%), while during the alpha and omicron periods, they were 5.6% and 5.1%, respectively. They also reported 42.7% of cases from the wild-type variant, otherwise than in our cohort [64]. The majority of our patients (69%) had no history of neurological diseases, and required special attention only when neurological or psychiatric disorders were a symptom of COVID-19. Such a possibility was pointed out by the CoroNerve Study Group in the U.K. [65], but this needs further investigation.

The differences in the course of COVID-19 between the five waves indicate the probable influence of different variants of SARS-CoV-2 on disease presentation. Until the second wave (October 2020 to January 2021), variants were not reported in Poland and SARS-CoV-2 sequencing was only performed occasionally. In the third wave (February to May 2021), the alpha (B.1.1.7) variant predominated and was reported to be associated with increased transmissibility (i.e., more efficient and rapid transmission). In January 2021, U.K. scientists reported evidence that suggested that the B.1.1.7 variant may be associated with an increased risk of death, but early reports found no evidence to suggest any effect on the severity of the disease [66]. In other countries, after the alpha variant announcement in December 2020, there were reports of increased admissions to hospital and more serious illnesses in children, indicating that the B.1.1.7. variant was more pathogenically infectious within this group [24]. Nevertheless, we found no evidence of more severe disease in children during the third wave, and we found that the B.1.1.7 variant did not result in an appreciably different clinical course than the original strain. The fourth wave was dominated by the B.1.617.2 delta variant, which was reported to have increased transmissibility. Many more patients were hospitalized and we observed more severe cases of COVID-19, but these were statistically insignificant. In the fifth wave, omicron (B.1.1.529, BA.1, BA.1.1, BA.2, BA.3, BA.4, and BA.5 lineages) dominated. The CDC announced that it caused a milder disease, although some people experienced a severe course, required hospitalization, and could have died from infection [67]. In this wave, we hospitalized 414 children and observed the shortest hospital stay.

In this study, the Bacillus Calmette–Guérin (BCG) vaccination was also considered to be a factor that influenced COVID-19 severity, because it was hypothesized that countries without widespread tuberculosis prevention policies had a higher percentage of severe cases (Italy, France, and Spain) than countries that adopted long-term widespread prevention (Japan, Denmark, and Korea). In Poland, antituberculosis BCG vaccination was obligatory, so in our pediatric study groups, over 95% of patients had been vaccinated. The lack of BCG vaccination was found in 2–4% of hospitalized children in different waves. We did not observe statistically significant differences in the number of hospitalized BCG-vaccinated and unvaccinated patients. However, various publications have described the results of the first association between BCG vaccination and COVID-19 cases, but these have concerned only adults [68,69].

Our study confirmed that the children had a much milder course of the virus and richer symptoms of COVID-19 compared to adults in all waves. The same has been reported in other studies [12,18,19,49,50,51].

### 4.3. Laboratory Findings

Only a few authors have compared the COVID-19 course in children between different waves of the pandemic. Most of them did not consider laboratory findings, while Murugan et al. did not find any significant differences in laboratory results (hemoglobin, total platelet count, creatinine, Alt, prothrombin time, partial thromboplastin time, D-dimer, and C-reactive protein) [9,10,13,14,15,24,25,26,70,71]. In our study, we found statistically significant differences in the first five waves of the pandemic in terms of CRP, blood platelets, and lactate dehydrogenase.

Our study has several limitations. During the first and second waves, primary care for COVID-19 patients was limited, so they were often referred to hospital. The Polish Ministry of Health’s recommendations about the rules for COVID-19 isolation and hospitalization changed in the subsequent waves, and this could have influenced the admission criteria and the length of hospitalization. Our experience with pediatric COVID-19 also expanded over the subsequent waves, which could also have influenced hospital admissions and the length of stay.

To the best of our knowledge, this is the first such large single-center study comparing the differences between the clinical course of pediatric COVID-19 in the first five waves of the pandemic.

## 5. Conclusions

Our findings confirmed that a life-threatening course of COVID-19 in children was relatively rare. However, children with pneumonia, dehydration from fever, gastrointestinal symptoms, and loss of smell and taste, as well as those with neurological symptoms, represented most of the patients requiring hospitalization.

The absolute number of hospitalizations was significantly higher in the fourth and fifth waves than in the first three waves. The clinical course of the disease changed between March 2020 and April 2022 due to the predominance of different SARS-CoV-2 variants.

## Figures and Tables

**Table 1:** Demographic characteristics of those COVID-19 pediatric patients hospitalized during the first five waves of the pandemic.

	First Wave *n* = 112	Second Wave *n* = 175	Third Wave *n* = 195	Fourth Wave *n* = 511	Fifth Wave *n* = 414	*p*-Value	Post Hoc Analysis
Male sex, *n* (%)	57 (50.9)	99 (56.6)	100 (51.3)	258 (50.7)	228 (55.1)	0.569 *	
Age, months Median (25th–75th percentile)	95.8 (17–130)	23.0 (5.7–85)	20.1 (5.4–55)	17.6 (5.3–68)	14.6 (5.4–43)	<0.001 ^#^	1 vs. 2: <0.001
							1 vs. 3: <0.001
							1 vs. 4: <0.001
							1 vs. 5: <0.001
Patients with chronic diseases, *n* (%)	39 (34.8)	37 (21.1)	63 (32.3)	111 (21.7)	107 (26.8)	0.06 *	
Immunocompromised patients, *n* (%)	2 (1.9)	6 (3.5)	5 (2.6)	4 (0.8)	4 (1.0)	0.064 *	
BCG vaccinated patients, *n* (%)	97 (96)	159 (98)	178 (98)	483 (96)	399 (97)	0.59 *	

* Chi-squared test; # Kruskal–Wallis ANOVA.

**Table 2:** Clinical characteristics of those COVID-19 pediatric patients hospitalized during the first five waves of the pandemic.

	First Wave *n* = 112	Second Wave *n* = 175	Third Wave *n* = 195	Fourth Wave *n* = 511	Fifth Wave *n* = 414	*p*-Value *
Fever, *n* (%)	76 (69)	117 (67)	146 (75)	347 (68)	296 (71)	0.327
Rhinitis, *n* (%)	24 (21)	48 (28)	78 (40)	266 (52)	193 (47)	<0.001
Cough, *n* (%)	42 (38)	62 (36)	119 (61)	326 (64)	221 (53)	<0.001
Dyspnea, *n* (%)	11 (10)	13 (7.4)	24 (12)	73 (14)	46 (11)	0.143
Vomiting, *n* (%)	7 (6.3)	37 (21)	27 (14)	91 (18)	126 (30)	<0.001
Diarrhea, *n* (%)	22 (20)	54 (31)	39 (20)	91 (18)	105 (25)	0.002
Anosmia, *n* (%)	3 (2.7)	7 (4)	3 (1.5)	8 (1.6)	54 (13)	<0.001
Ageusia, *n* (%)	2 (1.8)	5 (2.9)	3 (1.5)	10 (2)	54 (13)	<0.001
Neurologic symptoms, *n* (%)	15 (14)	19 (11)	9 (4.6)	52 (10)	38 (9.2)	0.077

* Chi-squared test.

**Table 3:** Laboratory findings in those COVID-19 pediatric patients hospitalized during the first five waves of the pandemic according to the Kruskal–Wallis and chi-squared tests.

	First Wave *n* = 112	Second Wave *n* = 175	Third Wave *n* = 195	Fourth Wave *n* = 511	Fifth Wave *n* = 414	*p*-Value	Post Hoc Analysis
CRP (mg/dL) (normal value = 0–5 mg/dL)	2.05 (1–9)	2.1 (1–12.6)	2.05 (1–14)	2.9 (1–10)	3.7 (1–11)	0.11 ^#^	
CRP >5 mg/dL	37 (38)	57 (38)	67 (36)	187 (38)	160 (41)	0.799 *	
Leukocytes (10^3^/μL) (normal value = 6–10 × 10^3^/μL)	6.4 (5–8.1)	8.2 (6–11.6)	8.9 (6.1–12.5)	8.0 (5.8–11)	7.75 (5.9–11.2)	<0.001 ^#^	1 vs. 2: <0.001 1 vs. 3: <0.001 1 vs. 4: <0.001 1 vs. 5: <0.001
Leukocytes (10^3^/μL)							
<4.5	18 (18)	19 (13)	19 (10)	59 (12)	35 (9)		
4.5–13.5	79 (78)	106(69)	131(69)	368 (75%)	301(37)		
>13.5	4 (4)	27 (18)	39 (21)	63 (13)	54 (14)		
Neutrophils (10^3^/μL) (normal value = 1.5–7 × 10^3^/μL)	2.89 (1.8–4.2)	2.62 (1.2–4.7)	2.62 (1.4–4.9)	2.59 (1.6–4.7)	2.7 (1.6–4.8)	0.925 ^#^	
Neutrophils (10^3^/μL)						0.016 *	
<1.0	7 (8)	26 (18)	29 (16)	65 (13)	45 (12)		
1.0–6.5	79 (87.6)	95 (64)	119 (68)	348 (74)	284 (73)		
>6.5	4 (4.4)	26 (18)	28 (16)	64 (13)	57 (15)		
Lymphocytes (10^3^/μL) (normal value = 2.5–8.5 × 10^3^/μL)	2.3 (1.5–3.2)	3.46 (2.1–5.3)	3.96 (2.4–6.4)	3.61 (2.1–5.7)	3.39 (1.7–5.7)	<0.001 ^#^	1 vs. 2: <0.001 1 vs. 3: <0.001 1 vs. 4: <0.001 1 vs. 5: <0.001
Lymphocytes (10^3^/μL)						<0.001 *	
<1.0	10 (11)	12 (8)	6 (3.4)	25 (5.3)	43 (11)		
1.0–7.0	78 (26)	117 (80)	139 (77)	381 (80.4)	297 (75)		
>7.0	3 (3)	17 (12)	33 (19)	68 (14.3)	48 (24)		
Blood platelets (10^3^/μL) (normal value = 210–560 × 10^3^/μL)	247 (192–298)	309 (247–411)	303 (243–360)	279 (215–363)	281 (222–351)	<0.001 ^#^	1 vs. 2: <0.001 1 vs. 3: <0.001 1 vs. 4: 0.005 1 vs. 5: 0.01
Blood platelets < 100 × 10³/μL	4 (4)	3 (2)	3 (1.6)	10 (2)	3 (0.8)	0.265 *	
Alanine transaminase (U/L) (normal value = 0–55 U/L)	16 (12–24)	18 (12–28)	19 (13–29)	20 (13–29)	22 (15–32)	<0.001 ^#^	5 vs. 1: <0.001 5 vs. 2: 0.015 5 vs. 3: 0.03 5 vs. 4: 0.017
Alanine transaminase >55 (U/L)	3 (3.2)	9 (6.3)	8 (4.5)	21 (4.5)	33 (8)	0.065 *	
Creatinine kinase (U/L) (normal value = 30–170 U/L)	80 (55–114)	84 (63–125)]	108 (71–157)	94 (65–146)	115 (83–168)	<0.001 ^#^	5 vs. 1: <0.001 5 vs. 2: <0.001 5 vs. 4: <0.001
Creatinine kinase							
170 (U/L)	5 (9.4)	12 (12.5)	31 (19)	66 (15.3)	82 (24)	0.005 *	
Lactate dehydrogenase (IU/L) (normal value = 125–220 (U/L)	238.5 (192–293)	268.5 (210–311)	286.0 (236–323)	272.0 (221–313)	293.0 (252–333)	<0.001 ^#^	1 vs. 3: <0.001 1 vs. 4: 0.023 1 vs. 5: <0.001 5 vs. 2: 0.001 5 vs. 4: <0.001
Lactate dehydrogenase >220 IU/L	51 (57)	101 (70)	148 (88)	316 (85)	280 (86)	<0.001 *	
D-dimers (ng/mL) (normal value = 0–500 ng/mL)	407 (256–694)	582 (377–1142)	471 (294–908)	559 (331–1082)	611 (397–1065)	<0.001 ^#^	1 vs. 2: 0.004 1 vs. 4: 0.004 1 vs. 5: <0.001
D-dimers > 500 ng/mL	33 (38)	80 (60)	72 (47)	205 (54)	184 (61)	<0.001 *	

* Chi-squared test, data are presented as *n* (%); ^#^ Kruskal–Wallis ANOVA, data are presented as the median (25th–75th percentile).

**Table 4:** COVID-19 outcomes in those hospitalized pediatric patients during the first five waves of the pandemic.

	First Wave *n* = 112	Second Wave *n* = 175	Third Wave *n* = 195	Fourth Wave *n* = 511	Fifth Wave *n* = 414	*p*-Value	Post Hoc Analysis
Oxygen therapy, *n* (%)	0	2 (1.1)	8 (4)	19 (4)	9 (2.2)	0.071 *	
Intravenous fluids, *n* (%)	24 (21)	62 (35)	18 (9)	243 (48)	243 (59)	<0.001 *	
Steroid therapy, *n* (%)	7 (6)	2 (1.1)	18 (9)	57 (11.2)	20 (4.8)	<0.001 *	
Antiviral therapy, *n* (%)	0	0	0	10 (1.9) (remdesivir—9, baricitinib—1)	2 (0.4) (baricitinib—2)	0.67 *	
Length of stay, *n* (days) Median (25th–75th percentile)	4 (2–6)	4 (3–5)	3 (2–6)	4 (3–5)	3 (2–4)	<0.001 ^#^	5 vs. 1: <0.001 5 vs. 2: <0.001 5 vs. 3: <0.001 5 vs. 4: <0.001

* Chi-squared test; ^#^ Kruskal–Wallis ANOVA.

**Table 5:** Final diagnoses during the first five waves of the pandemic.

	First Wave *n* = 134	Second Wave *n* = 212	Third Wave *n* = 247	Fourth Wave *n* = 694	Fifth Wave *n* = 57	* p * -Value
Upper respiratory tract infection	49 43.8%	99 56.9%	95 49.0%	277 54.6%	304 73.4%	<0.001
Lower respiratory tract infection	56 50.0%	53 30.5%	63 32.5%	162 32.0%	68 16.4%	<0.001
Gastroenterocolitis	10 8.9%	26 15.1%	32 16.5%	68 13.4%	101 24.4%	<0.001
Neurological diagnoses	5 4.5%	1 0.6%	16 8.2%	84 16.6%	41 9.9%	<0.001
Other	14 12.5%	33 19.1%	41 21.1%	103 20.3%	61 14.7%	0.07

*n*, number of diagnoses. Data are presented as *n* (%); *p*-value for chi-squared test.

**Table 6:** Lung imaging in COVID-19 pediatric patients hospitalized during the first five waves of the pandemic.

	First Wave *n* = 112	Second Wave *n* = 175	Third Wave *n* = 195	Fourth Wave *n* = 511	Fifth Wave *n* = 414
Chest X-ray, *n*	52	33	33	265	71
Positive X-ray, *n (%)*	24 (46)	25 (76)	33 (100)	149 (56)	51 (72)
Lung ultrasound, *n*	7	37	42	256	212
Positive lung ultrasound, *n (%)*	1 (14)	28 (76)	37 (88)	134 (52)	54 (18)
HRCT, *n*	0	0	2	11	0
Positive HRCT, *n* (%)	0	0	2 (100)	11 (100)	0

## Data Availability

The datasets used and analyzed during the current study are available from the corresponding author upon reasonable request.
